# Use of Successive Pharmacologic Hormone Suppression Testing for a Severe Presentation of Adolescent Polycystic Ovarian Syndrome: A Case Report

**DOI:** 10.1177/2324709619850215

**Published:** 2019-05-22

**Authors:** Sonalee Jaya Ravi, Melanie Cree-Green

**Affiliations:** 1 University of Colorado Anschutz Medical Campus, Aurora, CO, USA

**Keywords:** PCOS, adolescence, late-onset congenital adrenal hyperplasia, hirsutism

## Abstract

*Background*. Pathological causes of acne and hirsutism include polycystic ovarian syndrome (PCOS), congenital adrenal hyperplasia, and adrenal or ovarian tumors. PCOS is largely a clinical diagnosis and often simple laboratory testing can rule out more severe pathology. In more severe cases, determination of the correct diagnosis can require hormone suppression testing. In this article, we present a full sequence of hormone suppression testing and workup necessary to arrive at the ultimate diagnosis. *Case Presentation*. A 12-year-old normal weight (body mass index = 29th percentile), premenarchal female with Tanner III breast, Tanner V pubic hair presented with a 2.5-year history of severe hirsutism (Ferriman-Gallwey Score of 22), clitoromegaly, and deep voice. Successive hormone suppression and testing (ACTH stimulation testing, ovarian and adrenal imaging, dexamethasone-suppressed ACTH stimulation testing, and oral contraceptive therapy) was necessary to rule out congenital adrenal hyperplasia or a tumor and confirm PCOS. Metabolic testing, completed only after diagnosing PCOS, demonstrated insulin resistance. *Conclusions*. This patient had an extreme presentation of a common disorder. Her premenarchal status, elevated androgens, and virilization raised concern for non-PCOS pathology requiring sequential pharmacological hormone suppression testing and imaging for accurate diagnosis and appropriate treatment. The testing presented here is not novel, but we present the full sequence of testing and clinical results. This full sequence is rarely necessary for accurate diagnosis given clinical presentation and initial evaluation and, therefore, to our knowledge, has not been published. All providers caring for patients with PCOS should be familiar with this testing and its interpretation for severe cases that warrant extra attention.

## Background

Acne and hirsutism are common complaints in adolescent females. Acne peaks during puberty and is present in up to 85% of female youth.^1^ Hirsutism is quantified using the Ferriman-Gallwey Score. A score of 8 or higher in Caucasians is considered elevated.^2,3^ The most common cause of increased acne and hirsutism is normal pubertal changes with physiologic increased androgens. However, when either are more severe and accompanied by menstrual irregularities, the differential diagnosis should be expanded to other causes such as polycystic ovarian syndrome (PCOS), late-onset congenital adrenal hyperplasia (LOCAH), or an adrenal or ovarian tumor.^4,5^ PCOS is the most common cause of hirsutism with menstrual irregularity in adolescent women. It is a clinical diagnosis but is also a diagnosis of exclusion. Typically, simple laboratory testing will rule out LOCAH or a hormone-secreting tumor; however, in severe cases, such as that presented below, pharmacological suppression testing of adrenal or ovarian androgens may be required. Whereas this type of testing is not novel, the rarity of its use and the necessity in this case to employ hormonal suppression in succession for proper diagnosis will be presented.

## Case Presentation

A 12-year 7-month-old Caucasian female with no significant medical or family history was referred to pediatric endocrinology for progressively worsening acne, hirsutism, and a deep voice. She described excessive hair growth to the face, chest, abdomen, and back. She denied salt craving, increased thirst, or prolonged illnesses, and denied dizziness, headaches, or vision changes. She had breast development for about 2.5 years and was premenarchal. Review of her growth chart demonstrated linear growth acceleration around age 9 to 10 years with stable height for the past year and a body mass index at the 29th percentile.

**Figure 1.**
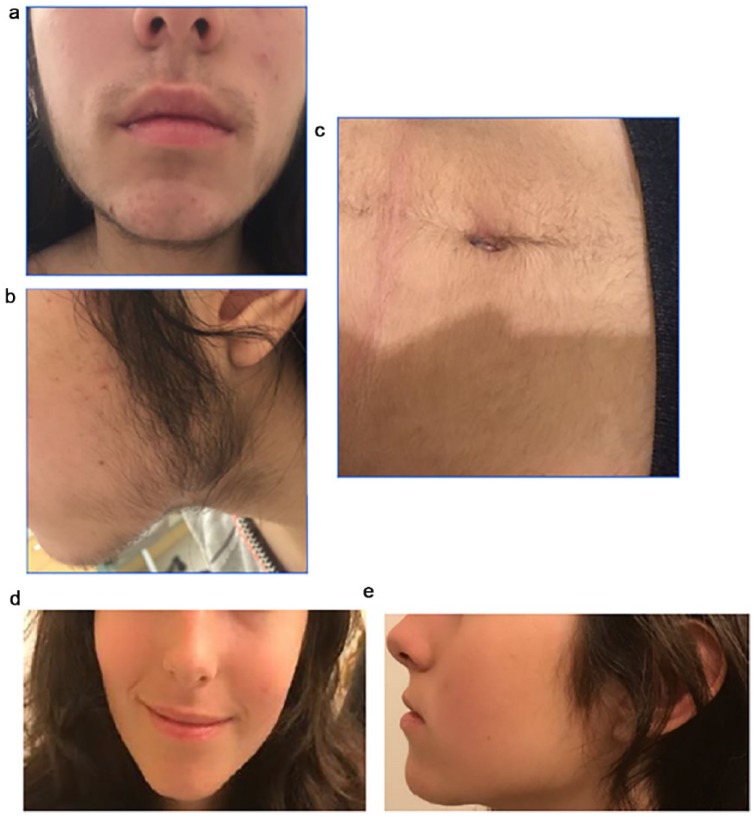
Hirsutism. (a and b) Face at presentation. (c) Abdomen at presentation. (d and e) Face at 6-month follow-up.

On physical examination, she was a normotensive, normocardic female with a deep voice. She had mild acne on the face and upper chest, significant hirsutism with a Ferriman-Gallwey Score of 22 (upper lip: 4, chin: 4, chest: 3, upper abdomen: 2, lower abdomen: 3, thighs: 3, lower back: 2, and upper back: 1) as seen in Figure 1a to c and grade 1 acanthosis nigricans to the neck. Her pubertal examination revealed mild clitoromegaly (5 cm long by 0.5 cm wide) with slightly enlarged labia minora that were larger than the labia majora, Tanner stage V pubic hair and axillary hair, and Tanner stage III breast development.

Initial laboratory values drawn at 2 pm are shown in Table 1 and demonstrated a 46 XX karyotype, and estrogen, prolactin, follicle stimulating hormone (FSH), and luteinizing hormone (LH) in the pubertal range with LH–FSH ratio greater than 2:1. Electrolytes and thyroid function were normal. Notably, the patient had an elevated androstenedione and testosterone, and a borderline elevated 17-hydroxyprogesterone. A bone age was read as 14 years (close to 2 standard deviations advanced).

**Table 1.** Hormone Measurements at Presentation, Before and After ACTH Stimulation Testing, After Dexamethasone Suppression and After ACTH Stimulation After Dexamethasone Testing at 3 Months and 6 Months Post Oral Contraceptive (OCP) Treatment.

Hormone Measurement (Norms, Units)	Initial Laboratory Values	Pre-ACTH Stimulation	Post-ACTH Stimulation	Post-Dexamethasone Suppression	ACTH Stimulation Post-Dexamethasone	Post 3 Months OCPs	Post 6 Months OCPs
Androgen pathway							
DHEA (<202 ng/dL)		**582**	585	193	**465**	74	287
DHEA-S (32-248 µg/dL)	208			94	95		
Androstenedione (28-230 ng/dL)	**413**	**734**	517	**438**	457	40	73
Total testosterone (6-50 ng/dL)	**121**	**148**	144	**162**	154	8.4	17
Free testosterone (0.9-6.8 pg/mL)	**21**						
Dihydrotestosterone (4-22 ng/dL)		17	17				
Glucocorticoid pathway							
17-OH pregnenolone (44-235 ng/dL)		**1047**	1362	53	**1023**	52	199
17-hydroxyprogesterone (<129 ng/dL)	**128**	**187**	236	108	**171**	16	38
11-desoxycortisol (12-158 ng/dL)		**177**	193	<10	123	20	29
Cortisol (2.5-25 µg/dL)	20.4	21	31	<1	23	39	22
Mineralocorticoid pathway							
Progesterone (10-856 ng/dL)		23	40	10	31	<10	38
11-Deoxycorticosterone (2-19 ng/dL)		**26**	39	3.6	**23**	2.4	6
Plasma renin activity (0.5-3.3 ng/mL)	2.69						
Non-adrenal							
Sex hormone–binding globulin (17-155 nmol/L)	32						
FSH (1.2-11 mIU/mL)	3.6						
LH (0.1-11 mIU/mL)	9.9						
Prolactin (1.9-25 ng/mL)	9						
Estradiol (7-170 pg/mL)	45.4	47	47				
Estrone (15-105 pg/mL)		89	71				

Abbreviations: FSH, follicle-stimulating hormone; LH, luteinizing hormone.

Boldface measures were interpreted as abnormal. Provided norms are for baseline, unstimulated values.

Due to the borderline elevated 17-hydroxyprogesterone, a 250-µg adrenocorticotrophic hormone (ACTH) stimulation test was performed. Nine am laboratory values prior to the stimulation test demonstrated multiple elevated steroids and androgens including 17-hydroxypregnenolone, 17-hydroxyprogesterone, 11-deoxycortisol, dehydroepiandrosterone (DHEA), androstenedione, and testosterone (Table 1). Additionally, she had a normal progesterone, estrone, and estradiol. There was minimal response of any axis to ACTH. This pattern of elevation and response was not consistent with a single enzyme defect causing CAH raising concern for an autologous androgen–producing tumor.

Based on results of the ACTH stimulation test, abdominal/pelvic imaging was obtained. Abdominal magnetic resonance imaging showed normal adrenal glands and no focal lesion. Pelvic ultrasound was notable for normal size ovaries (2.1 × 3.6 × 3.1 cm on the right and 2.5 × 2.7 × 2.7 cm on the left) with small peripheral cysts and normal blood flow and an anteverted uterus measuring 4.5 × 1.7 × 3.7 cm with a suboptimally visualized endometrial stripe.

Due to concern for a hormone-secreting mass too small to visualize, a dexamethasone-suppressed ACTH stimulation test was performed to further delineate the source of elevated androgens. Adrenal androgens (progesterone, deoxycorticosterone, 11-deoxycortisol, and cortisol) suppressed whereas the ovarian androgens (11-hydroxypregnenalone, 17-hydroxyprogesterone, DHEA, androstenedione, and testosterone) did not suppress, as shown in Table 1 indicating an ovarian source of androgen production. This led to the presumptive diagnosis of PCOS.

With a suspected PCOS, metabolic laboratory tests were performed and were normal except for elevated fasting and postprandial insulin concentrations as shown in Table 2. As the family’s primary treatment goal was to decrease hirsutism, the patient was started on 100 mg spironolactone daily and a 20 µg estrogen combined oral contraceptive. A lower dose estrogen was selected as she was premenarchal with incomplete breast development. Given her elevated postprandial insulin she was also started on 1000 mg of metformin twice a day, with a titration to full dose over a month.

**Table 2.** Metabolic Laboratory Values Prior to Initiation of Treatment.

Metabolic Measure (Norms, Units)	Value
Fasting glucose (60-100 mg/dL)	75
Hemoglobin A1C (4.5% to 5.7%)	5.3
AST (10-30 U/L)	23
ALT (10-30 U/L)	17
Total cholesterol (<170 mg/dL)	115
Triglycerides (<90 mg/dL)	61
HDL cholesterol (>45 mg/dL)	91
Non-HDL cholesterol (<120 mg/dL)	24
Fasting insulin (0.0-29.1 µIU/mL)	39.1
2-hour postprandial insulin (10.0-100 µIU/mL)	>300

Abbreviations: AST, aspartate transaminase; ALT, alanine transaminase; HDL, high-density lipoprotein.

After 3 months of multidrug treatment, the patient’s androgen (DHEA, androstenedione, and total testosterone) and glucocorticoid (17-hydroxyprogesterone, 17-hydroxypregnenolone, and 11-desoxycortisol) concentrations normalized confirming the presumption that the androgen source was the ovaries, and that she did not have an autonomously secreting mass. She had menarche with 2 days of very light bleeding rather than full menses and mild improvement in hirsutism and acne, with no changes in her voice. At that time, she was transitioned to a 30 µg estrogen containing combined oral contraceptive with a non-androgenic progesterone and then achieved full menses. At her 6-month follow-up, her hormone profile remained consistent with oral contraceptive therapy, and her hirsutism was dramatically improved (Figure 1d and e). She was also menstruating regularly. Her voice was still unchanged.

## Discussion and Conclusions

This case highlights an extreme presentation of a common syndrome and is unusual in that virilization and the high concentrations of androgens were not associated with LOCAH or an adrenal or ovarian tumor. Elevated androgens are common in young women. PCOS affects 6% to 10% of postpubertal females and is the most common cause of hyperandrogenemia in adolescent girls with the theca cells of the ovary being the source of elevation.^4^ Often PCOS is a clinical diagnosis made after ruling out the most pathological causes of hyperandrogenemia with a DHEAs, androstenedione, and 17-hydroxyprogesterone to evaluate for adrenal etiologies of hyperandrogenism. This patient, however, presented a more difficult case and required successive hormonal suppression testing to confirm the diagnosis and ensure proper diagnosis and treatment.

Clinically, the initial presentation of our patient was more extreme that typically seen for PCOS and suggestive of an alternative pathology. Her LH–FSH ratio fit the classic 2:1 pattern seen in PCOS and both were within the normal range for a Tanner III female. However, our patient was 12 years old and premenarchal with clitoromegaly, a total testosterone that was 2.4 times the upper limit of normal, and a free testosterone level that was 3 times the upper limit of normal. Classically, PCOS patients present between the ages of 15 and 25 years and rarely present with virilization. Furthermore, serum testosterone concentrations are seldom greater than twice the upper limit of normal for total testosterone and 4 times the upper limit of normal for free testosterone.^2,4^ Our patient’s age, premenarchal status, virilization, extreme hirsutism, and levels of elevated androgens led to a clinical concern for non-PCOS pathology and prompted extensive and sequential hormonal suppression evaluation do delineate the source of elevated androgen levels.

After PCOS, the next most common causes of hyperandrogenism are CAH and an adrenal or ovarian tumor. Only about 2% of patients with hyperandrogenemia have CAH but it is more likely in the presence of virilization.^6^ The most common form of CAH is 21-hydroxylase deficiency, characterized by an elevated 17-hydroxyprogesterone, serum testosterone, and androstenedione with significant elevation from ACTH stimulation.^6^ Our patient did not have a significant elevation in 17-hydroxyprogesterone after 250-µg ACTH stimulation testing. A less common cause of virilizing CAH is 11-β hydroxylase deficiency, characterized by hypertension, elevated androgens, and 11-deoxycortisol levels that are more than 3 times the upper limit for age-matched norms. Plasma renin activity and aldosterone are often suppressed as a result of salt and water retention induced by elevations of DOC. Unlike the heterozygotes with 21-OH deficiency, those with 11-OH deficiency often fail to show a rise in precursors following ACTH stimulation.^7^ However, an exuberant response was seen in those who had hirsutism. Nonetheless, her marked elevations in baseline androgens beyond that typically seen in PCOS were concerning for further pathology.

Despite not visualizing a tumor on abdominal/pelvic imaging, clinical suspicion remained for an adrenal or ovarian tumor, which would drastically change management prompting a dexamethasone-suppressed ACTH stimulation test.^6^ The test consists of giving a single dose of dexamethasone just before bedtime followed by a 0.25 mg ACTH stimulation test the next morning. Elevations in steroids can then be attributed to gonadal secretion or ACTH independent pathways. Non-suppression of 17-hydroxyprogesterone, DHEA, and androstenedione with suppression of cortisol is indicative of ovarian steroidogenesis. Conversely, suppression of both androgens and corticosteroids makes an ovarian source of androgens unlikely. Suppression of neither androgens nor glucocorticoids is evidence of an autonomously secreting, ACTH-independent adrenal tumor.^8^ In our patient, ovarian androgens did not suppress to expected levels, whereas adrenal androgens did, thereby indicating an ovarian source of elevated androgens. With the presumption of an ovarian source of hyperandrogenemia, estrogen can be used to confirm the androgen source. It is well documented that oral contraceptive therapy will suppress ovarian androgen production in PCOS, but not in the setting of a tumor.^9^ The exact duration of estrogen therapy for androgen suppression is not clear, but at least 5 to 7 days is needed to see acute changes. Our patient had normalization of androgen levels after 3 months of treatment confirming the presumption that the androgen source was the ovaries and that she did not have an autonomously secreting mass.

Metabolic syndrome is common in PCOS, even in normal weight girls.^10^ The patient had an elevated fasting and postprandial insulin concentrations.^11^ Insulin augments the effect of LH on the theca cell to produce excess androgens.^12^ LH stimulates the theca cells to convert cholesterol into androgens while FSH stimulates the granulosa cells to convert androgens into estrogen.^12^ Therefore, while not obese, treating this patient’s hyperinsulinemia was important in controlling her hyperandrogenism.

Treatment for PCOS should be personalized, based on both the patient’s stated goals and also tailored to maintain long-term metabolic health. She was treated with 100 mg spironolactone daily to reduce testosterone effects in the skin, 20 µg estrogen containing oral contraceptive to suppress ovarian androgens, and 1000 mg metformin twice daily to reduce hyperinsulinemia and suppress androgens. Her androgen concentrations, acne, and hirsutism greatly improved on treatment. Given her extreme presentation, she will likely need to stay on treatment control symptoms until she is interested in attempting to conceive.

This case demonstrates an extreme presentation of PCOS given the patient’s age, premenarchal status, severe hirsutism, virilization, and biochemical abnormalities provided clinical concern for more serious pathology. A diagnosis of PCOS was confirmed only after sequential hormonal suppression testing to rule out LOCAH or a tumor. Establishing the source and thereby cause of hyperandrogenemia was essential for the proper management and treatment. The ultimate diagnosis of PCOS led to metabolic testing, which revealed hyperinsulinemia despite the patient’s normal body mass index and therefore led to the initiation of metformin as adjunctive therapy, which may not have initially been considered.

In this article, we present the full sequence of testing (ACTH stimulation, dexamethasone-suppressed ACTH stimulation, and oral contraceptive therapy for final confirmation of diagnosis) and clinical results in its entirety for its educational benefit. This testing is necessary to arrive at the correct diagnosis in only a minority of cases with extreme presentations as was discussed. It is prudent that all providers caring for patients with PCOS across different disciplines be familiar with this testing to arrive at the correct diagnosis and therefore guide prognosis and management.
